# The relationship between diabetes-related knowledge and kidney disease knowledge, attitudes, and practices: a cross-sectional study

**DOI:** 10.1186/s12889-023-15390-8

**Published:** 2023-03-13

**Authors:** Asem Badran, Anas Bahar, Mohammed Tammam, Sami Bahar, Amani Khalil, Amer A. Koni, Sa’ed H. Zyoud

**Affiliations:** 1 Department of Medicine, College of Medicine and Health Sciences, An-Najah National University, 44839 Nablus, Palestine; 2 Department of Pediatric, An-Najah National University Hospital, 44839 Nablus, Palestine; 3 Faculty of Nursing, University of Jordan, Amman, Jordan; 4 Department of Clinical and Community Pharmacy, College of Medicine and Health Sciences, An-Najah National University, 44839 Nablus, Palestine; 5 Division of Clinical Pharmacy, Department of Hematology and Oncology, An-Najah National University Hospital, 44839 Nablus, Palestine; 6 Poison Control and Drug Information Center (PCDIC), College of Medicine and Health Sciences, An-Najah National University, 44839 Nablus, Palestine; 7 Clinical Research Center, An-Najah National University Hospital, 44839 Nablus, Palestine

**Keywords:** Diabetes mellitus, Chronic kidney disease, CKD screening index

## Abstract

**Background:**

Diabetes mellitus (DM) is one of the main noncommunicable diseases encountered in primary health care clinics. DM is considered one of the most common causes of chronic kidney disease (CKD). In this study, we aimed to assess the knowledge, attitudes, and practices (KAP) of patients with DM on the early detection and prevention of CKD, determine its relationship with other variables, and examine the relationship between KAP scores for the prevention and early detection of CKD and the Michigan Diabetic Knowledge Test.

**Methods:**

We collected data from 2 Nablus primary healthcare centers using a questionnaire that contains three sections: sociodemographic section, questions related to DM, and CKD screening index, which is formed of three scales. We used the Michigan Diabetic Knowledge Test (MDKT) to assess the knowledge of diabetic patients.

**Results:**

The study was carried out among 386 diabetic patients with a mean age of 57.62 ± 12.4 years (ranging from 28 to 90). The median (interquartile range) was 11 (8–14) for the knowledge scale, 56 (52–59) for the attitude scale, and 30 (26–33) for the practice scale. In the multiple linear regression, only patients under 55 years old (*p* = 0.012), with normal BMI (*p* = 0.030), high educational level (*p* < 0.001), high monthly income (*p* = 0.020), and MDKT test score (*p* = 0.007) were significantly associated with higher knowledge score. Furthermore, patients who were over or equal to 55 years old (*p* = 0.007), had a high monthly income (*p* = 0.016), used a single oral diabetic drug (*p* = 0.003), had a total number of medications less than 4 (*p* = 0.010), and had a high knowledge and MDKT test were significantly associated with a higher attitude score. Finally, a patient with normal BMI (*p* = 0.002), city residency (*p* = 0.034), high educational level (*p* = 0.003), less frequent tobacco use (*p* < 0.001), last HbA1c (*p* = 0.023) and greater knowledge, attitude, and MDKT score were significantly associated with better practices toward CKD prevention and early detection.

**Conclusion:**

Regarding KAP analysis, higher practice scores for the prevention and early detection of CKD were significantly associated with patients with normal BMI, being city residents, high educational level, less tobacco use, last HbA1c below 7, and higher knowledge, attitude, and MDKT score.

## Background

Diabetes mellitus (DM) is one of the main noncommunicable diseases encountered in primary care clinics. It is estimated that approximately 1 in 11 adults worldwide have diabetes, and 90% of them have type 2 DM [1]. The prevalence of type 2 DM in Palestine in 2010 was 15.3% and is expected to be 23.4% in 2030 [2], which is too high compared to the global prevalence of diabetes in 2015, which was 8.8%, and is expected to rise to 10.4% by 2040 [3]. However, there may be a significant decrease in the prevalence of DM if risk factors for it are controlled, especially obesity. It was suggested that a 2.8% reduction in diabetes prevalence could be achieved if obesity trends start to decline by 5% in 5 years [2].

Chronic kidney disease (CKD) is defined by Kidney Disease Improving Global Outcomes (KDIGO) and the National Kidney Foundation (NKF) Kidney Disease Outcomes and Quality Initiative (K/DOQI) as kidney damage (either functional or structural abnormalities of the kidneys) or a GFR < 60 ml/min/1.73 m^2^ for more than three months [4].

DM is considered one of the most common causes of CKD [5], which usually develops after long-standing poorly controlled DM. Nevertheless, many other factors can contribute to the development of CKD, including hypertension, urinary tract infections, nephrolithiasis, acute kidney injury (AKI), family history of CKD, old age, smoking, obesity, and nonsteroidal anti-inflammatory drugs (NSAIDs) [6].

In Palestine, there are a few studies on the prevalence of CKD among diabetic patients; one study showed that 23.6% of patients with type 2 DM in the North West Bank have CKD in different stages [7].

In general, there is a lack of awareness about possible risk factors, so the majority of CKD cases will not be recognized clinically or will be diagnosed at a later stage. Furthermore, it has been proven that the diagnosis of CKD will be delayed in patients with a positive attitude and solid knowledge and practices [8]. To avoid this, it is recommended to screen for CKD for early detection and treatment [9].

As DM and CKD still pose problems and it is possible to detect or delay the onset of CKD early, it is important to evaluate the knowledge, attitudes, and practice (KAP) of patients with DM related to the early detection and prevention of CKD. However, a literature review did not reveal studies among patients with DM in Palestine, so it is justified to establish a study on it.

We aimed to assess the KAP of patients with DM regarding early detection and prevention of CKD, determine its relationship with other variables, including clinical and sociodemographic factors, and examine the relationship between KAP scores for the prevention and early detection of CKD and the Michigan Diabetic Knowledge Test.

## Methods

### Study design

A cross-sectional study is used to assess the KAP of DM patients with regard to early detection and prevention of CKD using a newly developed index known as the CKD screening index. This index contains three scales regarding knowledge, attitudes, and practice, and its validity and reliability were ensured [8, 10].

### Study population

The study was held in Nablus, Palestine. Two primary healthcare centers were included, the Al-Makhfiya and Hiwara centers.

These primary care centers share a common fund provided by the Palestinian government. They are the main centers for primary care for most patients, including patients with DM, in the city of Nablus and surrounding villages, where patients are provided with essential medical care and drugs. The participant was chosen from registered patients and received treatment as diabetic patients in those primary care centers.

### Sample size and sampling procedure

The approximate number of patients who visited the diabetes clinics in the Nablus health center was 2000. The sample size was calculated using an online Raosoft sample size calculator (http://www.raosoft.com/samplesize.html). The minimum effective sample size was 323, assuming a 5% margin of error, a 95% confidence interval, and a response distribution of 50%. This study was conducted on 398 diabetic patients. We recruited participants who were conveniently available using the nonprobability sampling technique.

### Inclusion and exclusion criteria

Patients 18 years or older who could read and/or understand Arabic and had a diagnosis of DM for at least six months were included. However, we excluded patients with CKD diagnoses and conditions affecting their cognition, including stroke and mental illnesses. Additionally, patients with missing data were excluded from the final analysis.

### Data collection instrument

We used a questionnaire containing only one type of question that is close-ended. All subjects were interviewed in primary care centers by medical students who are familiar with the CKD screening index [8, 10].

It contained four sections. The first is the sociodemographic section, which contains a question on age, residency, sex, marital status, employment, educational level, monthly income, weight, and height. Body mass index (BMI) was calculated using the equation (weight “in kg”/height^2 (in m^^2^) and classified into underweight if less than 18.5, normal if greater than or equal to 18.5 to 24.9, overweight if greater than or equal to 25 to 29.9, obese if greater than or equal to 30, and morbid obese if greater than or equal to 40 [11]. Age was divided into two groups according to a previous study: 18–54 years and 55 years old and above [12].

The second section contained factors related to DM, such as duration of the disease, presence of comorbidities, type of therapy (monotherapy/combination, use of insulin), total number of medications, HbA1c and smoking status. The duration of DM was divided into two groups: below seven years and seven years or older [12]. The HbA1c reading was divided into two groups: below 7 and 7 or greater [12].

The third section contains the CKD screening index, which is divided into three different scales. The knowledge scale consists of 24 items regarding general knowledge of CKD, including its definition, risk factors, signs, symptoms, and complications. The attitudes scale, composed of 15 items, was used to assess the attitudes of diabetic patients toward CKD signs and symptoms and the ability to seek appropriate social and medical help related to their concerns. The practice score consists of 12 items that evaluate the health practices of each patient to prevent CKD. The validity and reliability of this screening index were examined and guaranteed [8] and used in its Arabic edition in another study [13].

The fourth section is the Michigan Diabetic Knowledge Test (MDKT). Its validity and reliability were evaluated [14] and used in its Arabic edition in previous studies [15–17]. We obtained developer permission to use it. We used the modified MDKT test, which contains the first 14 questions in the English edition of the online questionnaire (http://diabetesresearch.med.umich.edu/peripherals/profs/documents/svi/DKT2_with_answers.p-f) that are appropriate to assess knowledge of diabetes. It was a multiple-choice question that had only one single correct answer for each. It covers different aspects of diabetes, in which each question evaluates the patient’s knowledge of each. The correct answer was given one point, zero points for an incorrect answer, and the total score was 14. Therefore, more correct answers meant greater knowledge of DM.

### Ethical issues

All aspects of the study protocol, including access to and use of patient clinical information, were authorized by the *Institutional Review Boards (IRBs) of An-Najah National University* and the Palestinian Health Authority.

### Statistical analysis

Data were entered and analysed using the Statistical Package for Social Sciences program version 26 (IBM-SPSS 26). Data are expressed as the means ± SDs for continuous variables and as frequencies and percentages for categorical variables. The Kolmogorov–Smirnov test was performed to assess the normality of continuous data. The median (interquartile range (IQR)) and mean rank were used for variables that were not normally distributed. We used the Mann‒Whitney U test or Kruskal–Wallis test to detect and compare the differences between the medians of nonparametric data. The significance level was established at a *p* value of < 0.05. Additionally, we used multiple linear regression analysis for all univariate variables, which are significant in further evaluating the relationship between the patients’ KAP scores towards prevention of CKD, MDKT test scores, and the main clinical and sociodemographic variables. Internal consistency was assessed using Cronbach’s alpha for all CKD screening index subscales and MDKT.

## Results

### Sociodemographic and clinical characteristics

The total number of diabetic patients who participated and were interviewed was 398. Only 386 samples were included, 11 patients were excluded due to missing data, and one was newly diagnosed (less than six months). Sociodemographic and clinical characteristics are shown in Table 1. The mean age was 57.62 ± 12.4 years (range 28–90), and 58.5% of the subjects were ≥ 55 years old. Most of the subjects were male (*n* 211; 54.4%), married (75.4%), city residents (60.9%), employed (58.8%), and reached at least high school or more (62.7%). The mean BMI was 28.13 ± 3.86. DM was diagnosed in less than seven years in most patients (*n =* 223; 57.8%), the majority of them used only one oral drug for DM (*n* 240; 62.2%), and most patients used insulin (*n* = 240; 62.2%). Most patients had no chronic diseases other than DM (*n =* 181; 46.9%). The mean for the last HbA1c measurement was 8.18 ± 1.55 (Table 1).

**Table 1:** Sociodemographic and clinical characteristics of 386 patients with DM

Variable	Total: N = 386 (%)
**Age category (years)**	
< 55	160(41.5)
≥ 55	226 (58.5)
**Gender**	
Male	211(54.7)
Female	175 (45.3)
**BMI category**	
Normal	84 (21.8)
Overweight	181 (46.9)
Obese	121 (31.3)
**Residency**	
Refugee camp	32 (8.3)
Village	119 (30.8)
City	235 (60.9)
**Marital status**	
Married	291 (75.4)
Widow	52 (13.5)
Divorced	19 (4.9)
Unmarried	24 (6.2)
**Educational level**	
No formal education	45 (11.7)
Elementary school	99 (25.6)
High school	137 (35.5)
Collage/University	105 (27.2)
**Employment**	
Employed	227 (58.8)
Unemployed	159 (41.2)
**Monthly income (NIS** ^**a**^ **)**	
Low (< 2000)	177 (45.9)
Moderate (2000–5000)	160 (41.5)
High (> 5000)	49 (12.7)
**Smoking**	
Yes	165 (42.7)
No	221 (57.3)
**Duration of DM (years)**	
< 7	223 (57.8)
≥ 7	163 (42.2)
**Number of oral medications for DM**	
Mon therapy	240 (62.2)
Multi therapy	71 (18.4)
No oral medications	75 (19.4)
**Use of insulin**	
Yes	240 (62.2)
No	146 (37.8)
**Last HbA1c**	
< 7	71 (18.4)
≥ 7	315 (81.6)
**Comorbidities**	
Yes	205 (53.1)
No	181 (46.9)
**Total number of chronic diseases (other than DM)**	
0	181 (46. 9)
1	110 (28.5)
2	67 (17.4)
3	28 (7.2)
**Total number of medications other than DM medications**	
< 4	303 (78.5)
≥ 4	83 (21.5)

**Abbreviations**: BMI: body mass index, NIS: New Israeli shekel, HbA1c: hemoglobin A1c

^**a**^ 1NIS equals 0.31US Dollar

Approximately 47.9% (*n* = 185) of patients had hypertension, 8.5% (*n* = 33) had ischemic heart disease, 8.3% (n = 32) had congestive heart failure, 8% (*n* = 31) had rheumatoid arthritis, 5.4% (*n* = 21) had asthma, and 6.7% (*n* = 26) had other diseases, including inflammatory bowel disease (n = 10) and COPD (*n* = 3).

### CKD screening index ‘KAP’ and MDKT

#### Knowledge

The participants’ mean score on the knowledge scale was 11.27 ± 4.6, and the correct responses ranged from (0–24). The median and interquartile range of knowledge was 11 (8–14). The Cronbach’s alpha was 0.788, indicating good internal consistency. Furthermore, only 41.5% of diabetic patients knew that CKD is irreversible. Most patients (62.2%) did not know that smoking increased the risk of CKD. Furthermore, a higher percentage of patients did not realize that CKD can affect their concentration (76.6%) or their sleep pattern (68.9%), that it can cause muscle aches and pain, especially during the night (70.7%), or that it can cause skin dryness and itchiness (73.6%). More than half of the patients (55.7%) did not know that a procedure requiring contrast injection as cardiac catheterization could affect their kidney function.

Finally, only approximately 32.9% of the patients knew that CKD has five stages and that each stage requires special medical care, but most patients (58%) did not know that the final stage of CKD would require lifelong dialysis.

#### Attitude

The participants’ mean score on the attitude scale was 55.61 ± 5.52, ranging from 39 to 72. The median of the attitude scale was 56 (52–59). The Cronbach’s alpha coefficient was 0.648, indicating an acceptable level of internal consistency [18, 19].

Participants were generally more likely to agree or strongly agree on positive attitudes or beliefs toward CKD. For example, most patients (89.1%) visited a healthcare specialist if they felt any signs or symptoms of CKD. Furthermore, most patients agree or strongly agree that it is important for health to exercise and eat a balanced diet (88.6%), and regular check-ups with their doctor will make them less concerned about their health (72.3%).

Finally, 78.2% of patients agree or strongly agree that their healthcare provider has to give them more information about CKD.

#### Practice

The mean score on the practice scale was 29.59 ± 5.19, ranging from 13 to 42. The median of the attitude scale was 30 (26–33). The Cronbach’s alpha coefficient was acceptable, 0.747.

Generally, most of the patient’s responses were against positive practices most of the time or always and toward negative practices such as smoking or drinking alcohol. However, only 31.1% responded mostly or always to a balanced diet, and 17.3% responded to regular physical exercise.

### Michigan diabetic knowledge test (MDKT)

The mean scores on the MDKT test were 6.7 ± 2.75, ranging from 1 to 13. The median was 7 (5–9). The internal consistency was 0.601, indicating an acceptable Cronbach’s alpha coefficient [18, 19].

There was an obvious lack of knowledge about diabetes, and the diet among diabetic patients (55.4%) had an incorrect answer about the food that was highest in carbohydrates. (63.5%) did not know the effect of unsweetened fruit juice on blood glucose. A total of 52.1% did not know that HbA1c reflected their blood glucose over the last three months.

In terms of Spearman correlation between the three KAP scales and the MDKT test, there was a positive correlation between the practice score and the level of knowledge of CKD (r = 0.292; *p* < 0.001). Furthermore, knowledge about CKD positively correlated with knowledge about DM assessed by the MDKT test (r = 0.215; *p* < 0.001). Furthermore, the practice score was associated with a moderate positive correlation with knowledge of DM assessed by the MDKT test (r = 0.151; *p* = 0.003). On the other hand, the attitude score was associated with a moderate negative correlation with knowledge about DM assessed by the MDKT test (r = -0.318; *p* < 0.001).

### Characteristics of patients that associated with the knowledge score

In the bivariate analysis, higher knowledge scores were significantly associated with patients less than 55 years of age, normal BMI, city resident, unmarried, high educational level, employed, high income, no comorbidities, and used less than four medications other than DM drugs (Table 2). However, in the analysis with multiple linear regression, only patients under 55 years old (*p* = 0.012), normal BMI (*p* = 0.030), high educational level (*p* < 0.001), high income (*p* = 0.020) and higher MDKT test score (*p* = 0.007) were significantly associated with higher knowledge score (Table 3).

**Table 2:** The median knowledge score of 386 diabetic patients related to the prevention and early detection of chronic kidney disease

Variable	Total: N = 386 (%)	Median knowledge score ^a^ [Q1-Q3]	Mean rank	*P* value ^b^
**Age category (years)**				
< 55	160(41.5)	12 (10–16)	224.37	**< 0.001** ^**c**^
≥ 55	226 (58.5)	10(8–14)	171.65	
**Gender**				
Male	211(54.7)	11(8–14)	183.43	0.051^c^
Female	175 (45.3)	11(9–15)	205.64	
**BMI category**				
Normal	84 (21.8)	12(9–14)	203.43	**0.043** ^**d**^
Overweight	181 (46.9)	11(9–15)	202.94	
Obese	121 (31.3)	11(8–13)	172.48	
**Residency**				
Refugee camp	32 (8.3)	12(9–15)	206.39	**< 0.001** ^**d**^
Village	119 (30.8)	9(7–13)	151.85	
City	235 (60.9)	12(10–15)	212.84	
**Marital status**				
Married	291 (75.4)	11(9–14)	197.47	**0.002** ^**d**^
Widow	52 (13.5)	10(5–13)	143.64	
Divorced	19 (4.9)	12(9–17)	220.89	
Unmarried	24 (6.2)	13(11–15)	231.65	
**Educational level**				
No formal education	45 (11.7)	10(6–12)	139.73	**< 0.001** ^**d**^
Elementary school	99 (25.6)	9(7–11)	133.02	
High school	137 (35.5)	12(9–14)	204.78	
Collage/University	105 (27.2)	13(11–17)	258.85	
**Employment**				
Employed	227 (58.8)	12(9–15)	211.58	< 0**.001**^**c**^
Unemployed	159 (41.2)	10(7–14)	167.69	
**Monthly income (NIS** ^**e**^ **)**				
Low (Less than 2000)	177 (45.9)	11(8–14)	171.88	**< 0.001** ^**d**^
Moderate (2000–5000)	160 (41.5)	12(9–14)	202.65	
High (More than 5000)	49 (12.7)	13(10–17)	241.71	
**Smoking**				
Yes	165 (42.7)	11(9–14)	186.79	0.306^c^
No	221 (57.3)	11(8–15)	198.51	
**Duration of DM (years)**				
< 7	223 (57.8)	11(9–15)	199.07	0.250^c^
≥ 7	163 (42.2)	11(8–14)	185.88	
**Number of oral medications for DM**				
Mon therapy	240 (62.2)	11(8–14)	189.25	0.108^d^
Multi therapy	71 (18.4)	11(8–14)	182.70	
No oral medications	75 (19.4)	12(10–15)	217.33	
**Use of insulin**				
Yes	240 (62.2)	11(8–14)	188.89	0.297^c^
No	146 (37.8)	11(9–15)	201.07	
**Last HbA1c**				
< 7	71 (18.4)	12(10–15)	214.82	0.074^c^
≥ 7	315 (81.6)	11(8–14)	188.70	
**Comorbidities**				
Yes	205 (53.1)	10(8–13)	175.51	**0.001** ^**c**^
No	181 (46.9)	12(10–15)	213.87	
**Total number of chronic diseases (other than DM)**				
0	181 (46.9)	12(10–15)	213.87	**< 0.001** ^**d**^
1	110 (28.5)	11(9–14)	193.45	
2	67 (17.4)	11(8–14)	179.34	
≥ 3	28 (7.2)	8(6–10)	95.89	
**Total number of medications other than DM medications**				
< 4	303 (78.5)	12(9–15)	201.59	**0.006** ^**c**^
≥ 4	83 (21.5)	10(8–13)	163.96	

**Abbreviations**: BMI: body mass index, NIS: New Israeli shekel, HbA1c: hemoglobin A1c

^**a**^ Knowledge scale contains 24 items (range 0–24, the higher the score, the better knowledge)

^**b**^ cut-off level of significance was 0.05

^**c**^ Mann‒Whitney U test was used to detect statistical significance

^**d**^ Kruskal‒Wallis test was used to detect statistical significance

^**e**^ 1NIS equals 0.31US Dollar

**Table 3:** Characteristics of diabetic patients that are associated with the knowledge score related to prevention and early detection of chronic kidney disease in multiple linear regression

Variables ^a^	Unstandardized coefficients (B)	Standardized coefficients (Beta)	P value ^b^	95% confidence interval for B
**Constant**	9.122		**< 0.001**	6.393 to 11.851
**Age category (years)**	-1.177	− 0.126	**0.012**	-2.088 to − 0.265
**BMI category**	− 0.631	− 0.099	**0.030**	-1.201 to − 0.061
**Residency** ^c^	0.372	0.052	0.275	− 0.297 to 1.040
**Marital status** ^c^	− 0.188	− 0.022	0.647	− 0.624 to 0.388
**Educational level** ^c^	1.347	0.285	**< 0.001**	0.837 to 1.857
**Employment** ^c^	0.402	0.546	0.462	− 0.671 to 1.475
**Monthly income (NIS** ^**e**^ **)**	0.918	0.137	**0.020**	0.148 to 1.687
**Comorbidities** ^c^	− 0.766	− 0.083	0.327	-2.301 to 0.770
**Total number of chronic diseases (other than DM)**	− 0.832	− 0.170	0.090	-1.774 to 0.128
**Total number of medications other than DM medications**	− 0.081	− 0.007	0.907	-1.447 to 1.284
**MDKT test**	0.230	0.137	**0.007**	0.064 to 0.396
**R**: 0.504; **R Square**: 0.254; **Adjusted R Square**: 0.232; **Std. Error of the Estimate**: 4.04045				

**Abbreviations**: BMI: body mass index, NIS: New Israeli shekel, HbA1c: hemoglobin A1c, MDKT: Michigan Diabetes Knowledge Test

^**a**^ Multiple linear regression was done on each factor with a p value < 0.05

^**b**^ cut-off level of significance was 0.05

^**c**^ dichotomous variable is used to represent the nominal variables

### Characteristics of patients that associated with attitude score

In bivariate analysis, higher attitude scores were significantly associated with patients who were 55 years old or older, refugees in camps, high income, patients with DM less than seven years duration, using a single oral drug for DM, and less than four medications other than DM drugs (Table 4). Furthermore, in multiple linear regression, we found that patients who were older than or equal to 55 years of age (*p* = 0.007) had a high monthly income (*p* = 0.016), used a single oral diabetic drug (*p* = 0.003), had a total number of medications less than 4 (*p* = 0.010) and had high knowledge and MDKT test scores, which were significantly associated with a higher attitude score (Table 5).

**Table 4:** The median score for the attitude of 386 diabetic patients related to the prevention and early detection of chronic kidney disease

Variable	Total: N = 386 (%)	Median knowledge score [Q1-Q3]	Mean rank	*P* value
**Age category (years)**				
< 55	160(41.5)	55(50–59)	180.10	0.**047**^**c**^
≥ 55	226 (58.5)	56(52–59)	202.99	
**Gender**				
Male	211(54.7)	55(51–59)	187.13	0.217^c^
Female	175 (45.3)	56(52–59)	201.19	
**BMI category**				
Normal	84 (21.8)	55(51–60)	191.16	0.947^d^
Overweight	181 (46.9)	56(52–59)	195.45	
Obese	121 (31.3)	56(52–59)	192.21	
**Residency**				
Refugee camp	32 (8.3)	59(56–61)	246.34	**0.003** ^**d**^
Village	119 (30.8)	57(52–59)	204.88	
City	235 (60.9)	55(51–58)	180.54	
**Marital status**				
Married	291 (75.4)	56(52–59)	200.30	0.189^d^
Widow	52 (13.5)	56(50–59)	176.25	
Divorced	19 (4.9)	52(50–61)	177.74	
Unmarried	24 (6.2)	55(49–57)	160.90	
**Educational level**				
No formal education	45 (11.7)	55(51–59)	179.62	0.340^d^
Elementary school	99 (25.6)	55(52–59)	187.28	
High school	137 (35.5)	57(52–59)	207.12	
Collage/University	105 (27.2)	55(52–59)	187.54	
**Employment**				
Employed	227 (58.8)	55(51–59)	189.48	0.396^c^
Unemployed	159 (41.2)	56(52–59)	199.25	
**Monthly income (NIS** ^**a**^ **)**				
Low (Less than 2000)	177 (45.9)	55(51–59)	178.69	**< 0.001** ^**d**^
Moderate (2000–5000)	160 (41.5)	55.5(52–59)	191.65	
High (More than 5000)	49 (12.7)	59(55–63)	253.03	
**Smoking**				
Yes	165 (42.7)	56(52–60)	196.47	0.651^c^
No	221 (57.3)	56(52–59)	191.28	
**Duration of DM (years)**				
< 7	223 (57.8)	57(52–60)	205.96	**0.010** ^**c**^
≥ 7	163 (42.2)	55(51–58)	176.46	
**Number of oral medications for DM**				
Mon therapy	240 (62.2)	57(53–60)	213.55	**< 0.001** ^**d**^
Multi therapy	71 (18.4)	55(51–58)	172.16	
No oral medications	75 (19.4)	52(50–57)	149.55	
**Use of insulin**				
Yes	240 (62.2)	56(51–59)	190.89	0.555^c^
No	146 (37.8)	56(52–59)	197.79	
**Last HbA1c**				
< 7	71 (18.4)	56(51–58)	187.76	0.631^c^
≥ 7	315 (81.6)	56(52–59)	194.79	
**Comorbidities**				
Yes	205 (53.1)	55(51–59)	189.43	0.444^c^
No	181 (46.9)	56(52–59)	197.11	
**Total number of chronic diseases (other than DM)**				
0	181 (46.9)	56(52–59)	198.11	0.730^d^
1	110 (28.5)	56(52–59)	195.50	
2	67 (17.4)	55(51–59)	182.95	
≥ 3	28 (7.2)	54(51–60)	181.05	
**Total number of medications other than DM medications**				
< 4	303 (78.5)	56(52–59)	203.55	**0.001** ^**c**^
≥ 4	83 (21.5)	53(51–57)	156.82	

**Abbreviations**: BMI: body mass index, NIS: New Israeli shekel, HbA1c: hemoglobin A1c

^**an**^ Attitude scale is 15 items on a 5-point Likert-type scale (range 15–75, the higher score, the better attitudes)

^**b**^ Cut-off level of significance was 0.05

^**c**^ Mann‒Whitney U test was used to detect statistical significance

^**d**^ Kruskal‒Wallis test was used to detect statistical significance

^**e**^ 1NIS equals 0.31US Dollar

**Table 5:** Characteristics of diabetic patients that are associated with attitude score related to prevention and early detection of chronic kidney disease in multiple linear regression

Variables ^a^	Unstandardized coefficients (B)	Standardized coefficients (Beta)	P value ^b^	95% Confidence interval for B
**Constant**	57.564		**< 0.001**	55.404 to 59.724
**Age category (years)**	1.542	0.138	**0.007**	0.426 to 2.658
**Residency** ^c^	− 0.621	− 0.072	0.135	-1.437 to 0.195
**Monthly income (NIS** ^**e**^ **)**	0.951	0.119	**0.016**	0.177 to 1.725
**Duration of DM (years)**	− 0.002	0.000	0.997	-1.110 to 1.105
**Types of medications for DM** ^c^	-1.008	− 0.145	**0.003**	-1.680 to − 0.336
**Total number of medications other than DM medications**	-1.706	− 0.127	**0.010**	-2.999 to − 0.414
**Knowledge score**	0.171	0.143	**0.005**	0.051 to 0.292
**MDKT test**	− 0.525	− 0.261	**< 0.001**	− 0.722 to − 0.329
**R**: 0.438; **R Square**: 0.192; **Adjusted R Square**: 0.175; **Std. Error of the Estimate**: 5.02076				

**Abbreviations**: NIS: new Israeli shekel, DM: diabetes mellitus, MDKT: Michigan Diabetes Knowledge Test

^**a**^ Multiple linear regression was done on each factor with a p value < 0.05

^**b**^ cut-off level of significance was 0.05

^**c**^ Dummy coding was used to represent nominal variables

^**D**^ Knowledge scale contains 24 items (range 0–24, the higher the score, the better knowledge)

### Characteristics of patients associated with the practice score

In the bivariate analysis, a higher practice score was significantly associated with patients who were less than 55 years of age, had normal BMI, were city residents, were unmarried, had a high educational level, were employed, had a high income, were smokers, and had HbA1c less than 7 (Table 6). In an analysis with multiple linear regression, we found that normal BMI (*p* = 0.002), city residency (*p* = 0.034), high educational level (*p* = 0.003), smoking status (*p* < 0.001), last HbA1c (*p* = 0.023) and higher knowledge, attitude, and MDKT score were significantly associated with better practices toward the prevention and early detection of CKD (Table 7).

**Table 6:** The median score for the practice of 386 diabetic patients related to the prevention and early detection of chronic kidney disease

Variable	Total: N = 386 (%)	Median knowledge score [Q1-Q3]	Mean rank	*P* value
**Age category (years)**				
< 55	160(41.5)	30(27–34)	210.33	**0.012** ^**c**^
≥ 55	226 (58.5)	29(26–32)	181.58	
**Gender**				
Male	211(54.7)	30(26–33)	194.67	0.821^c^
Female	175 (45.3)	30(26–33)	192.09	
**BMI category**				
Normal	84 (21.8)	30(27–35)	214.92	0.**001**^**d**^
Overweight	181 (46.9)	30(27–33)	204.26	
Obese	121 (31.3)	28(25–32)	162.54	
**Residency**				
Refugee camp	32 (8.3)	30(25–32)	176.55	**0.001** ^**d**^
Village	119 (30.8)	29(24–32)	164.16	
City	235 (60.9)	30(27–34)	210.67	
**Marital status**				
Married	291 (75.4)	30(26–33)	199.56	**< 0.001** ^**d**^
Widow	52 (13.5)	27(25–30)	140.09	
Divorced	19 (4.9)	27(25–31)	165.82	
Unmarried	24 (6.2)	34(28–36)	257.69	
**Educational level**				
No formal education	45 (11.7)	25(22–29)	104.67	**< 0.001** ^**d**^
Elementary school	99 (25.6)	29(26–32)	176.45	
High school	137 (35.5)	30(26–33)	192.60	
Collage/University	105 (27.2)	32(29–35)	248.82	
**Employment**				
Employed	227 (58.8)	31(27–34)	216	**< 0.001** ^**c**^
Unemployed	159 (41.2)	28(25–32)	161.38	
**Monthly income (NIS** ^**a**^ **)**				
Low (Less than 2000)	177 (45.9)	29(25–32)	167.87	**< 0.001** ^**d**^
Moderate (2000–5000)	160 (41.5)	30(27–33)	202.51	
High (More than 5000)	49 (12.7)	32(30–37)	256.66	
**Smoking**				
Yes	165 (42.7)	31 (27–35)	218.55	**< 0.001** ^**c**^
No	221 (57.3)	29 (26–32)	174.79	
**Duration of DM (years)**				
< 7	223 (57.8)	30(26–33)	195.49	0.682^c^
≥ 7	163 (42.2)	29(26–33)	190.78	
**Number of oral medications for DM**				
Mon therapy	240 (62.2)	30(26–33)	194.13	0.114^d^
Multi therapy	71 (18.4)	28(26–32)	172.75	
No oral medications	75 (19.4)	31(27–33)	211.12	
**Use of insulin**				
Yes	240 (62.2)	30(26–33)	200.41	0.118^c^
No	146 (37.8)	29(26–33)	182.14	
**Last HbA1c**				
< 7	71 (18.4)	32(29–34)	235.06	**0.001** ^**c**^
≥ 7	315 (81.6)	29(26–33)	184.13	
**Comorbidities**				
Yes	205 (53.1)	30(26–33)	189.60	0.464^c^
No	181 (46.9)	30(26–34)	197.92	
**Total number of chronic diseases (other than DM)**				
0	181 (46.9)	30(26–34)	197.92	0.767^d^
1	110 (28.5)	30(27–33)	195.21	
2	67 (17.4)	30(25–33)	182.97	
≥ 3	28 (7.2)	29(27–32)	183.39	
**Total number of medications other than DM medications**				
< 4	303 (78.5)	30(27–33)	196.50	0.312^c^
≥ 4	83 (21.5)	29(25–33)	182.56	

**Abbreviations**: BMI: body mass index, NIS: New Israeli shekel, HbA1c: hemoglobin A1c

^**A**^ The practices scale contains 12 items (the core ranged from 12 to 48; the higher score, the better practice)

^**b**^ Cut-off level of significance was 0.05

^**c**^ Mann‒Whitney U test was used to detect statistical significance

^**d**^ Kruskal‒Wallis test was used to detect statistical significance

^**e**^1 new Israeli shekel (NIS) equals 0.31US Dollar

**Table 7:** Characteristics of diabetic patients that are associated with practice scores related to prevention and early detection of chronic kidney disease in multiple linear regression

Variables ^a^	Unstandardized coefficients (B)	Standardized coefficients (Beta)	P value ^b^	95% Confidence interval for B
**Constant**	21.226		**< 0.001**	15.414 to 27.038
**Age category (years)**	0.216	0.021	0.672	− 0.785 to 1.217
**BMI category**	-1.005	− 0.140	**0.002**	-1.644 to − 0.366
**Residency** ^c^	0.802	0.100	**0.034**	0.061 to 1.544
**Marital status** ^c^	0.331	0.054	0.248	− 0.232 to 0.893
**Educational level** ^c^	0.876	0.165	**0.003**	0.294 to 1.459
**Employment** ^c^	− 0.013	− 0.001	0.984	-1.229 to 1.204
**Monthly income (NIS** ^**e**^ **)**	0.721	0.096	0.109	− 0.162 to 1.604
**Smoking**	-1.806	− 0.172	**< 0.001**	-2.799 to − 0.813
**Last HbA1c**	-1.390	− 0.104	**0.023**	-2.587 to − 0.194
**Knowledge score**	0.220	0.196	**< 0.001**	0.107 to 0.334
**Attitude score**	0.093	0.099	**0.042**	0.003 to 0.183
**MDKT test**	0.217	0.115	**0.027**	0.025 to 0.409
**R**: 0.527; **R Square**: 0.278; **Adjusted R Square**: 0.254; **Std. Error of the Estimate**: 4.48175				

**Abbreviations**: BMI: body mass index, NIS: new Israeli shekel, HbA1c: hemoglobin A1c, MDKT: Michigan Diabetes Knowledge Test

^**a**^ Multiple linear regression was done on each factor with a p value < 0.05

^**b**^ cut-off level of significance was 0.05

^**C**^ dummy coding was used to represent nominal variables

### Characteristics of the patient that are associated with the MDKT score

In the bivariate analysis, the characteristics significantly associated with higher MDKT scores were male gender, city residents, unmarried patients, high education level, employed, with moderate to high income, no insulin use or oral medication for DM, duration of less than seven, years, HbA1c less than seven and presence of a single comorbid disease (Table 8). In an analysis with multiple linear regression, we found that city residency (*p* = 0.001), a high education level (*p* < 0.001), employment status (*p* = 0.005), monthly income (*p* < 0.001), longer duration of DM (*p* < 0.001) and no insulin use (*p* < 0.001) were significantly associated with a better score on the MDKT test (Table 9).

**Table 8:** Michigan Diabetes Knowledge Test of 386 patients with DM

Variable	Total: N = 386 (%)	Median knowledge score [Q1-Q3]	Mean rank	*P* value
**Age category (years)**				
< 55	160(41.5)	7(5–9)	200.70	0.283^c^
≥ 55	226 (58.5)	7(4–9)	188.40	
**Gender**				
Male	211(54.7)	7(5–9)	204.56	**0.032** ^**c**^
Female	175 (45.3)	6(4–8)	180.17	
**BMI category**				
Normal	84 (21.8)	6(4–8)	170.24	0.073^d^
Overweight	181 (46.9)	7(4–9)	196.24	
Obese	121 (31.3)	7(5–9)	205.55	
**Residency**				
Refugee camp	32 (8.3)	6(4–7)	139.77	**< 0.001** ^**d**^
Village	119 (30.8)	6(4–8)	167.61	
City	235 (60.9)	7(5–9)	213.93	
**Marital status**				
Married	291 (75.4)	7(5–9)	199.06	**0.043** ^**d**^
Widow	52 (13.5)	6(4–8)	162.93	
Divorced	19 (4.9)	6(4–7)	157.32	
Unmarried	24 (6.2)	7(6–9)	220.96	
**Educational level**				
No formal education	45 (11.7)	6(4–8)	157.10	**< 0.001** ^**d**^
Elementary school	99 (25.6)	6(4–8)	177.70	
High school	137 (35.5)	6(4–8)	171.90	
Collage/University	105 (27.2)	8(7–10)	252.18	
**Employment**				
Employed	227 (58.8)	7(5–9)	207.08	**0.004** ^**c**^
Unemployed	159 (41.2)	6(4–8)	174.12	
**Monthly income (NIS** ^**a**^ **)**				
Low (Less than 2000)	177 (45.9)	7(5–9)	196.02	**< 0.001** ^**d**^
Moderate (2000–5000)	160 (41.5)	7(5–9)	208.75	
High (More than 5000)	49 (12.7)	4(4–7)	134.61	
**Smoking**				
Yes	165 (42.7)	7(4–9)	187.88	0.390^c^
No	221 (57.3)	7(5–9)	197.69	
**Duration of DM (years)**				
< 7	223 (57.8)	6(4–8)	175.6	**< 0.001** ^**c**^
≥ 7	163 (42.2)	7(6–9)	217.99	
**Number of oral medications for DM**				
Mon therapy	240 (62.2)	6(4–9)	179.63	**0.003** ^**d**^
Multi therapy	71 (18.4)	7(5–9)	204.99	
No oral medications	75 (19.4)	8(6–9)	227.03	
**Use of insulin**				
Yes	240 (62.2)	6(4–8)	174.87	**< 0.001** ^**c**^
No	146 (37.8)	7(6–9)	224.13	
**Last HbA1c**				
< 7	71 (18.4)	7(6–10)	227.49	**0.004** ^**c**^
≥ 7	315 (81.6)	7(4–9)	185.84	
**Comorbidities**				
Yes	204 (52.8)	7(4–9)	196.27	0.602^c^
No	182 (47.2)	7(5–9)	190.36	
**Total number of chronic diseases (other than DM)**				
0	181 (46.9)	7(5–9)	190.36	**0.031** ^**d**^
1	110 (28.5)	7(5–9)	210.95	
2	67 (17.4)	7(5–9)	194.76	
≥ 3	28 (7.2)	5(3–8)	142.20	
**Total number of medications other than DM medications**				
< 4	303 (78.5)	7(4–9)	189.50	0.176^c^
≥ 4	83 (21.5)	7(5–9)	208.08	

**Abbreviations**: BMI: body mass index, NIS: New Israeli shekel, HbA1c: hemoglobin A1c, DM: diabetes mellitus

^**A**^ Knowledge scale contains 24 items (range 0–24, the higher the score, the better knowledge)

^**b**^ Cut-off level of significance was 0.05

^**c**^ Mann‒Whitney U test was used to detect statistical significance

^**d**^ Kruskal‒Wallis test was used to detect statistical significance

^**e**^1 new Israeli shekel (NIS) equals 0.31 US Dollar

**Table 9:** Characteristics of diabetic patients that were associated with Michigan Diabetic Knowledge related to prevention and early detection of chronic kidney disease in multiple linear regression

Variables ^a^	Unstandardized coefficients (B)	Standardized coefficients (Beta)	P value ^b^	95% Confidence interval for B
**Constant**	5.259		**< 0.001**	4.058 to 6.461
**Gender**	− 0.412	− 0.075	0.141	− 0.962 to 0.137
**Residency** ^c^	0.635	0.149	**0.001**	0.245 to 1.024
**Marital status** ^c^	− 0.163	− 0.050	0.287	− 0.463 to 0.138
**Educational level** ^c^	0.618	0.219	**< 0.001**	0.318 to 0.917
**Employment** ^c^	− 0.966	− 0.173	**0.005**	-1.633 to − 0.299
**Monthly income (NIS** ^**e**^ **)**	-1.061	− 0.266	**< 0.001**	-1.521 to-0.602
**Duration of DM**	1.000	0.180	**< 0.001**	0.476 to 1.524
**Types of oral medications**	0.328	0.095	0.068	− 0.024 to 0.677
**Use of insulin**	1.312	0.232	**< 0.001**	0.778 to 1.849
**Last HbA1c**	− 0.362	− 0.051	0.270	-1.006 to 0.282
**Total number of chronic diseases other than DM**	− 0.068	− 0.024	0.626	− 0.343 to 0.207
**R**: 0.513; **R Square**: 0.263; **Adjusted R Square**: 0.242; **Std. Error of the Estimate**: 2.39498				

**Abbreviations**: NIS: new Israeli shekel, DM: diabetes mellitus, HbA1c: hemoglobin A1c

^**A**^ multiple linear regression was done on each factor with a p value < 0.05

^**b**^ cut-off level of significance was 0.05

^**c**^ Dummy coding was used to represent nominal variables

## Discussion

CKD has become a serious global health problem [20], and it is important to have good and reliable data on the knowledge, attitudes, and practices of patients at risk of developing CKD. However, CKD could be prevented or its progression could be slowed by using a three-level strategy. First, we should begin with primary prevention, which includes public education and modified risk factors. The second level, secondary prevention, includes screening and slowing disease progression. The third level, tertiary prevention, includes optimal management of patients with CKD.

Few studies about KAP among diabetic patients are available on the early detection and prevention of CKD. These include research done in Jordan that developed and used the CKD screening index, a reliable, valid, and generalizable index used to assess the KAP in CKD prevention and early detection [10]. Thus, we used this index in Nablus, Palestine, to assess how healthy practices to prevent or detect CKD early are affected by good knowledge and positive attitudes.

In our study, there was a great lack of knowledge about CKD and its signs, symptoms, and risk factors, as the mean knowledge score was 11.27 out of 24 compared to a study conducted in Jordan that showed a mean score of 19.27 out of 24 using the same screening index [10] and a mean score of 18.55 out of 30 in another study in Palestine [13]. We found that a higher practice score was significantly associated with being less than 55 years old, having a normal BMI, being a city resident, having a higher income and educational level, and having HbA1c of less than seven. In regression analysis, we found that a higher practice score for CKD prevention and early detection was significantly associated with normal BMI, being a city resident, high educational level, less tobacco use, last HbA1c below 7, higher knowledge, good attitude, and higher MDKT. The better practices among these patients can be attributed to a higher knowledge of diabetes, as seen in the MDKT test, and a higher knowledge about possible signs, symptoms, and risk factors for developing CKD. The higher the knowledge about diabetes evaluated on the MDKT test, the more likely the patient will comply with his medications and improve self-care and awareness of possible complications of DM, including CKD [21].

Our study found an obvious lack of knowledge about diabetes and its diet among diabetic patients using the MDKT test, similar to a study in Saudi Arabia showing that only 21.6% of patients had good knowledge about diabetes [21]. The mean score was low at 47.8% (6.7 out of 14 with a standard deviation of 2.75), which is close to a study conducted in the United Arab Emirates (UAE) that showed mean scores of 55% in 2016, 55.5% in 2001 and 68.2% in 2006 [22].

Poor knowledge can challenge attitudes and practices, as a previous study showed that knowledge about cardiovascular health improves attitudes and practices [23]. Therefore, if patients had sound knowledge about diabetes, they would have good attitudes and practices regarding secondary prevention, such as chronic kidney diseases. Specifically, human beings’ practices depend on their attitudes regarding each behavior, and these attitudes are built on what they know about the outcomes [24].

In addition, there were no relationships between the age of the patient and his knowledge of diabetes. However, another study showed a slight decrease in knowledge with increasing age [22]. We also found that patients with higher education levels are more likely to have a better knowledge about diabetes than in a study in the UAE [22]. Multiple linear regression shows that a city resident with a high income and educational level using insulin therapy with a longer duration of DM is significantly associated with better knowledge about DM. A similar study using the MDKT test also showed that patients with a longer duration of DM, insulin use, and higher educational levels are significantly associated with better knowledge about DM [21].

As we said, most patients will visit their doctors if they have any signs or symptoms of CKD. Unfortunately, due to the lack of knowledge about the signs and symptoms of CKD, this will not be applicable. Therefore, this attitude will not benefit the early detection of CKD without adequate education about CKD.

Finally, incorrect assumptions about risk factors, signs and symptoms, disease stages, and related management plans can explain why patients present late for medical help. Therefore, it is encouraged to have a good education program and screening protocols to prevent and detect CKD. This approach improved medical outcomes among patients diagnosed with CKD [25].

### Strengths and limitations

This study has a strong point. It is one of the few studies related to KAP to prevent and detect CKD early. To our knowledge, this is the first study in Palestine specifically for diabetic patients. Furthermore, it is a multicenter study, making it more representative of diabetic patients in Nablus. In addition, medical students familiar with these scales were given the forms of data collection and scales, which will reduce the risk of misunderstood or missing data. Finally, the KAP was measured using a screening index developed, tested, and validated by Khalil and Abdalrahim in 2014, making the results convenient and reliable [10].

Despite the strengths, there are limitations regarding the generalizability of these results. For example, the sample was restricted only to patients who regularly followed up in the Nablus primary health care center at the Al-Makhfia and Hiwara primary health care centers and was limited to patients who visited these outpatient clinics, meaning that it may not represent patients who cannot reach the clinics due to serious illnesses. In addition, although we have attempted to minimize the recall and selection biases, they are still limitations of the current study. Furthermore, only close-ended questions were included in the questionnaire, which would decrease the ability to identify the underlying reasons for certain results. Another limitation was that cross-sectional studies could not generally determine the cause-effect and temporal relationships between sociodemographic and clinical characteristics and KAP scores. Furthermore, the sample size was small, which might not be sufficient to identify the real differences that are considered statistically significant in some sociodemographic characteristics and clinical factors. Additionally, some variables, such as the type of diabetes medication that the patient used, were not analysed. Finally, we did not have information on the patient’s current renal function, which could have helped to develop and assess the relationship between the current renal status and their level of KAP score on early detection and prevention.

## Conclusion

Patients generally have poor knowledge about DM, its diet, and its complications, making them susceptible to complications and a poor disease prognosis. We found that a higher practice score for prevention and early detection was significantly associated with normal BMI, being a city resident, high educational level, less tobacco use, last HbA1c below seven, and higher knowledge, attitude, and MDKT. Although patients have some information about CKD, most have incorrect assumptions about signs and symptoms and risk factors related to CKD. Therefore, they are unaware of the behaviors that can protect against CKD and the importance of early detection. Therefore, it is strongly recommended that patients improve their knowledge of the signs and symptoms and risk factors for CKD, and educating them about incorrect practices may increase the risk of CKD. This can be achieved through national educational programs focusing on patients with an increased risk of CKD as DM in this case and on the general population beginning early in the school population to establish baseline knowledge and positive attitudes and practices. It is also recommended to provide a screening protocol for high-risk patients for early detection of CKD to reduce or halt the progression of the disease.

## Data Availability

Due to privacy, the data sets used and/or analysed during the current study are available from the corresponding author on reasonable request. This manuscript is part of a Doctor of Medicine graduation project submitted to An-Najah National University. The abstract was published as part of self-archiving in institutional repositories (university repository: https://repository.najah.edu/handle/20.500.11888/16078?show=full).
